# Standardization and consensus in the development and application of bone organoids

**DOI:** 10.7150/thno.105840

**Published:** 2025-01-01

**Authors:** Jian Wang, Xiao Chen, Ruiyang Li, Sicheng Wang, Zhen Geng, Zhongmin Shi, Yingying Jing, Ke Xu, Yan Wei, Guangchao Wang, Chongru He, Shiwu Dong, Guohui Liu, Zhiyong Hou, Zhidao Xia, Xinglong Wang, Zhou Ye, Fengjin Zhou, Long Bai, Hongbo Tan, Jiacan Su

**Affiliations:** 1 Department of Orthopedics, Xinhua Hospital Affiliated to Shanghai Jiao Tong University School of Medicine, Shanghai, 200092, China.; 2 Trauma Orthopedics Center, Xinhua Hospital Affiliated to Shanghai Jiao Tong University School of Medicine, Shanghai 200092, China.; 3 Institute of Musculoskeletal Injury and Translational Medicine of Organoids, Xinhua Hospital Affiliated to Shanghai Jiao Tong University School of Medicine, Shanghai, 200092, China.; 4 Institute of Translational Medicine, Shanghai University, Shanghai, 200444, China.; 5 National Center for Translational Medicine SHU Branch, Shanghai University, Shanghai, 200444, China.; 6 Department of Orthopedics, Shanghai Zhongye Hospital, Shanghai, 200941, China.; 7 Sixth People's Hospital Affiliated to Shanghai Jiao Tong University School of Medicine, Shanghai 200233, China.; 8 Department of Biomedical Materials Science, College of Biomedical Engineering, Third Military Medical University, Chongqing, 400038, China.; 9 Union Hospital Affiliated to Tongji Medical College, Huazhong University of Science and Technology, Wuhan 430022, China.; 10 Department of Orthopaedic Surgery, Third Hospital of Hebei Medical University, Shijiazhuang, 050051, China.; 11 Institute of Life Science, College of Medicine, Swansea University, Swansea, SA2 8PP, UK.; 12 Department of Pharmacology and Toxicology, University of Arizona, Tucson, AZ, 85721, USA.; 13 Applied Oral Sciences and Community Dental Care, Faculty of Dentistry, The University of Hong Kong, Hong Kong, 999077, China.; 14 Department of Orthopedics, Honghui Hospital, Xi'an Jiao Tong University, Xi'an, 710000, China.; 15 Department of Orthopedics, 920th Hospital of Joint Logistics Support Force of Chinese PLA, Kunming, 650032, China.

**Keywords:** bone organoids, organoid development, disease modeling, bone tissue engineering, standardization, *in vitro* modeling

## Abstract

Organoids, self-organized structures derived from stem cells cultured in a specific three-dimensional (3D)* in vitro* microenvironment, have emerged as innovative platforms that closely mimic* in vivo* cellular behavior, tissue architecture, and organ function. Bone organoids, a frontier in organoid research, can replicate the complex structures and functional characteristics of bone tissue. Recent advancements have led to the successful development of bone organoids, including models of callus, woven bone, cartilage, trabecular bone, and bone marrow. These organoids are widely utilized in establishing bone-related disease models, bone injury repair, and drug screening. However, significant discrepancies remain between current bone organoids and human skeletal tissues in terms of morphology and functionality, limiting their ability to accurately model human bone physiology and pathology. To address these challenges and promote standardization in the construction, evaluation, and application of bone organoids, we have convened experts and research teams with substantial expertise in the field. By integrating existing research findings, this consortium aims to establish a consensus to guide future research and application of bone organoids.

## 1. Introduction

In clinical practice, bone repair and regeneration present complex and challenging issues involving numerous common orthopedic diseases and pathological conditions 1. According to the International Osteoporosis Foundation, osteoporosis causes approximately 8.9 million fractures annually worldwide, translating to an osteoporotic fracture every three seconds 2. In the European Union, the annual number of osteoporotic fractures was about 4.28 million in 2019, with this number expected to rise due to an aging population ​^3^. Repairing bone defects requires prolonged treatment, and the outcomes are often unsatisfactory. Traditional repair methods, such as autografts and allografts, are limited by factors such as donor scarcity, immune rejection, and insufficient regeneration capacity 4. The complexity of bone drug screening is compounded by the inadequacy of existing two-dimensional (2D) cell cultures and animal models to fully replicate the physiological environment of human bone tissue 5. Concurrently, bone tumors and genetic bone diseases, such as osteogenesis imperfecta, impose a significant health burden on patients, and current treatment options yield limited results 6. This necessitates more advanced research tools and treatment strategies. In this context, organoid technology is seen as a promising innovative approach that can offer new possibilities for bone disease research and therapeutic development 7.

Organoids are miniature tissue structures formed from stem or progenitor cells under specific three-dimensional (3D) culture conditions, capable of mimicking the complex structures and physiological functions of specific organs *in vitro* 8, 9. Since their inception, organoid technology has achieved significant progress in the study of various tissues, such as intestinal, hepatic, and pulmonary organoid models, which have been widely used in disease mechanism research and drug screening 10-12. Bone organoids, as a new branch of organoid technology, provide a more physiologically relevant method to study bone tissue growth, development, and pathological changes 13. Compared to traditional 2D cultures and animal models, bone organoids better reflect the 3D structure, cell interactions, and changes in pathological conditions of bone tissue 14. This ability to closely simulate physiological states makes bone organoids ideal tools for drug screening, disease research, and regenerative medicine research, potentially accelerating the development and clinical translation of new therapies 15, 16.

To date, the development and application of bone organoid technology remain in their early stages, with limited reports available in the literature. This consensus paper outlines the potential directions for the construction, evaluation, and application of bone organoids, while systematically addressing the technical challenges and opportunities. By summarizing current research findings, we aim to provide clear guidance for future bone organoid studies and promote their broad application in bone disease treatment and regenerative medicine. This paper not only offers researchers a comprehensive perspective but also lays the foundation for the standardized development and application of bone organoids.

## 2. What are bone organoids?

### 2.1 The origin of organoids

**Figure 1:**
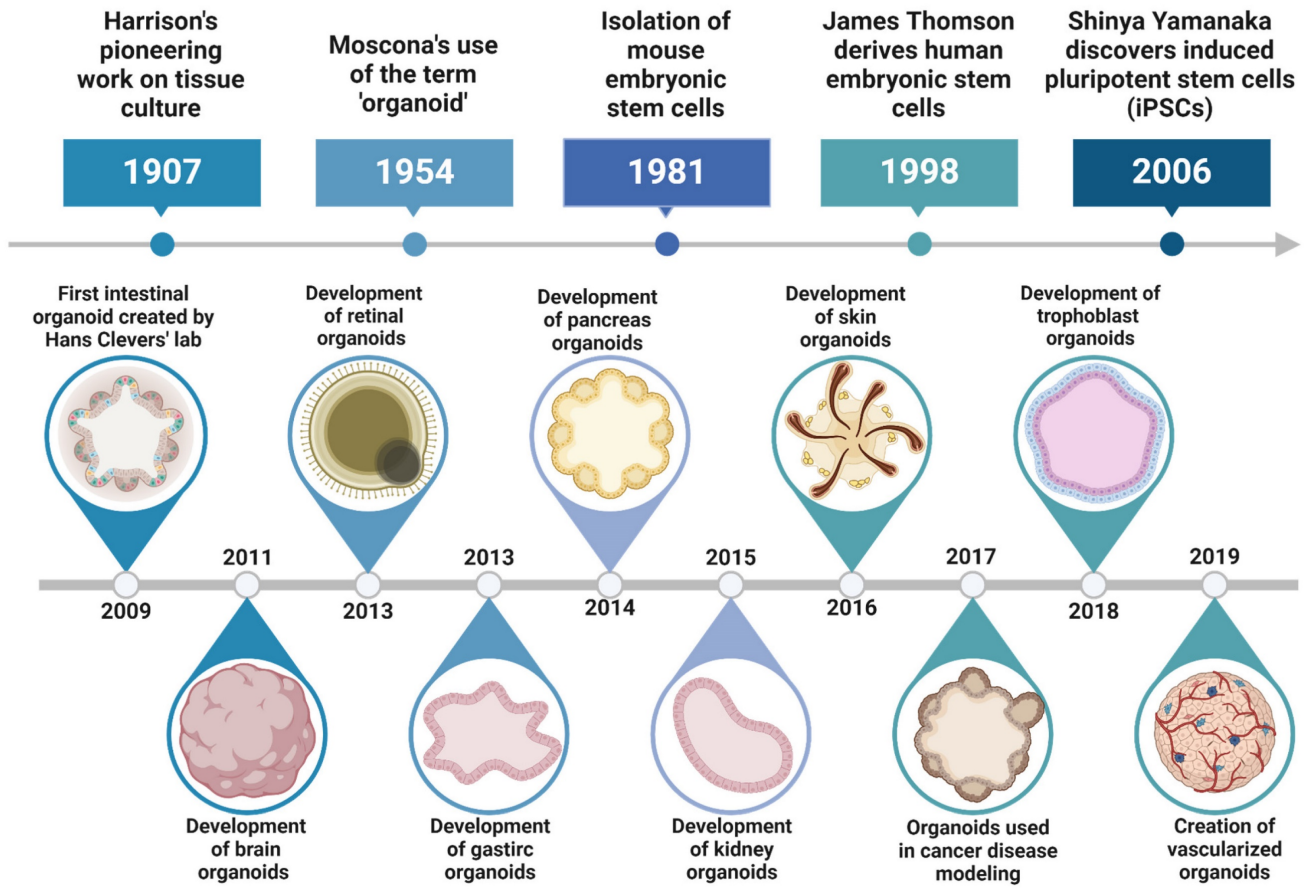
** Key milestones in organoid research.** Timeline of major advancements in organoid development, from early tissue culture in 1907 to recent models like vascularized organoids in 2019.

Organoids are miniature tissue structures formed through the self-organization of embryonic and adult mammalian stem cells under specific 3D culture conditions, enabling them to partially replicate the complex cellular composition and physiological characteristics of original organs, and reflecting key structural and functional properties of organs such as the kidney, lung, gut, brain, and retina 17. The concept of organoids originated from the recognition of the self-organizing capabilities of stem cells. Significant milestones include the discovery and research of stem cells, with hematopoietic stem cells identified in the 1960s and subsequent discoveries of neural, intestinal, and skin stem cells 18. The successful isolation and culture of embryonic stem cells (ESCs) from mice in 1981 and humans in 1998 by scientists such as Martin Evans, Matthew Kaufman, and James Thomson provided powerful tools for studying stem cell differentiation and self-organization 19. In 2006, Shinya Yamanaka's team reprogrammed adult cells into induced pluripotent stem cells (iPSCs), addressing ethical issues and expanding research cell sources. In 2009, Hans Clevers' team achieved a significant breakthrough by culturing functional small intestinal organoids from mouse intestinal stem cells, demonstrating that stem cells can self-organize into structures resembling *in vivo* organs under 3D culture conditions 20. This led to the successful cultivation of organoids for various human organs, such as the brain, liver, pancreas, and kidneys, revolutionizing disease research, drug screening, and personalized medicine 21 **(Figure 1)**. The continuous optimization of culture media and conditions has enhanced the complexity and functionality of organoids, bringing them closer to their* in vivo* counterparts and expanding their potential in biomedical research and clinical applications.

### 2.2 Characteristics of organoids

Organoids are structures formed through the self-organization of stem cells or progenitor cells under specific 3D culture conditions, exhibiting cellular composition, structure, and function similar to *in vivo* organs 22. They have the following notable characteristics. Multicellularity, as organoids consist of various types of cells that interact to form complex 3D structures. This multicellular composition allows organoids to more accurately simulate the microenvironment and physiological functions of *in vivo* organs 23. Self-organization, under specific culture conditions, organoids can self-organize and differentiate to form structures resembling *in vivo* organs. This self-organization capability enables organoids to serve as a valuable model for studying cell behavior and tissue development 24. Functionality, as organoids possess certain functions of *in vivo* organs, such as metabolism, secretion, and the ability to respond to external stimuli 25. Intestinal organoids can mimic the absorptive function of the intestinal epithelium, while brain organoids can exhibit the electrical activity of neural networks.

### 2.3 Definition and concept of bone organoids

**Figure 2:**
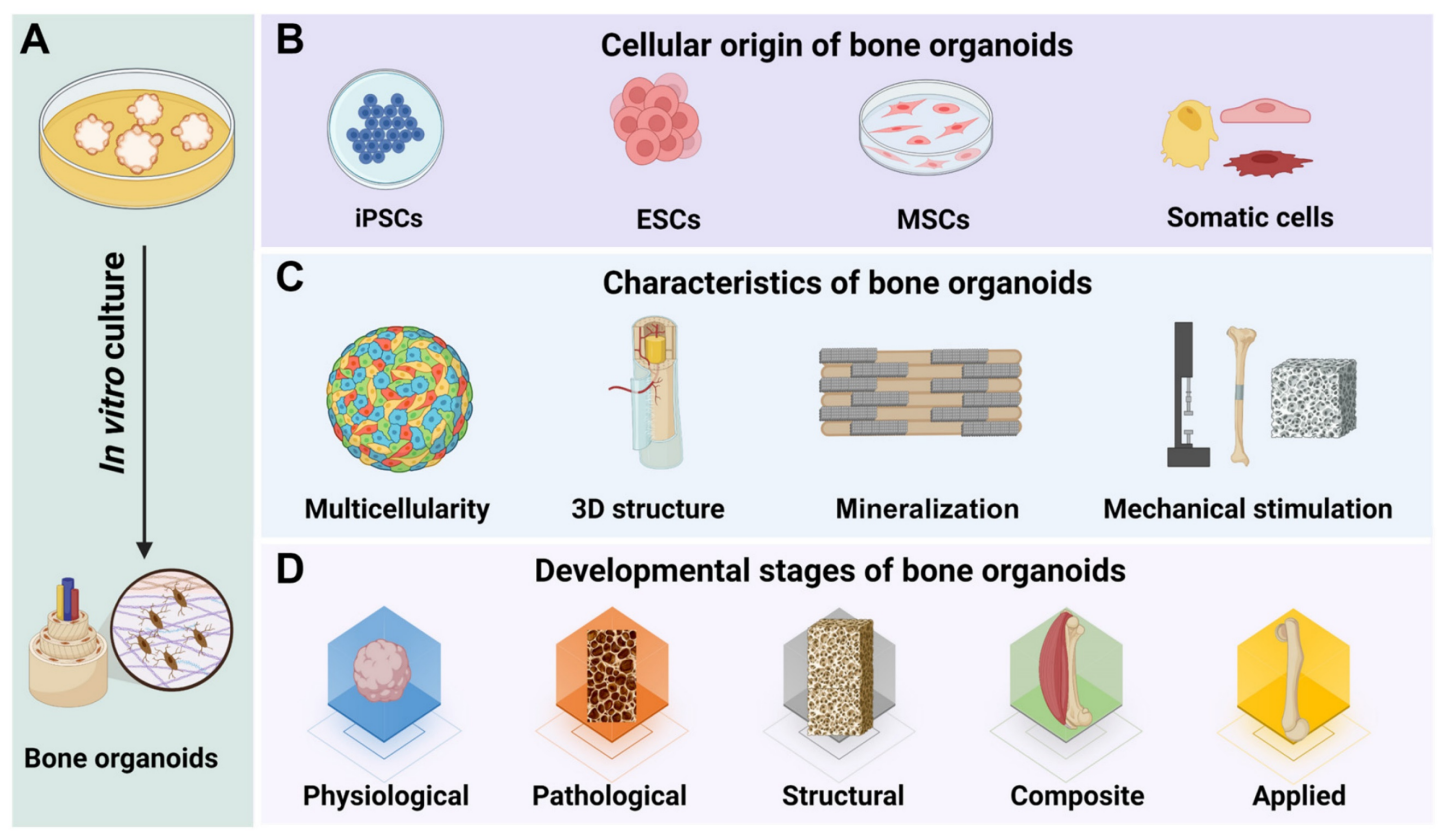
** Overview of bone organoids: definition, cellular origin, key characteristics, and classification by developmental stage. (A)** Bone organoids are complex, 3D tissue constructs cultured *in vitro*, replicating the structure and function of bone. **(B)** The cellular sources of bone organoids include iPSCs, ESCs, MSCs, and somatic cells. **(C)** Bone organoids are characterized by multicellularity, a 3D bone tissue structure, mineralization capacity, and mechanical support. **(D)** Our team's perspective on the developmental stages of bone organoids.

Bone organoids are 3D, miniaturized, and simplified versions of bone tissue that are created *in vitro* using stem cells or progenitor cells 15 **(Figure 2)**. These organoids are engineered to mimic the structure, function, and cellular complexity of natural bone tissue, including key features like mineralization and vascularization. Bone organoids are grown in 3D cultures, allowing them to develop more realistic tissue architecture compared to traditional 2D cell cultures. They consist of multiple cell types found in natural bone, such as osteoblasts (bone-forming cells), osteoclasts (bone-resorbing cells), and osteocytes (mature bone cells). Bone organoids undergo a process of mineralization, where they deposit calcium phosphate minerals to form a rigid, bone-like matrix 26. Advanced bone organoids may develop blood vessel-like structures, enhancing their similarity to real bone tissue and allowing for better nutrient and waste exchange. These organoids exhibit functional characteristics of bone, such as the ability to support hematopoiesis (blood cell formation) and respond to biochemical signals that regulate bone growth and remodeling 27.

### 2.4 Bone organoids: distinct models derived from human skeletal tissue

Bone organoids, while distinct models derived from human skeletal tissue, are not equivalent to actual human bone. Bone organoids and human bone tissue exhibit key differences in cellular composition, mineralization, biomechanical properties, vascularization, and repair mechanisms. Bone organoids, generated *in vitro* or via 3D modeling, lack the complex microenvironment and endogenous signaling seen in human bone. Their mineralization is incomplete, and their mechanical strength is significantly lower—e.g., the ultimate compressive strength of human femoral bone is 168-355 MPa, compared to just 5-30 MPa in organoids 26, 28. Additionally, while bone organoids can form microvessels with external growth factors, their vascular density remains much lower than that of human bone marrow (25-30%) 27. Bone organoids also display slower and less effective repair processes, failing to fully replicate the dynamic remodeling and healing mechanisms of human bone 29. While promising for modeling bone function and diseases, these discrepancies limit their ability to fully substitute human bone tissue. While promising for modeling bone function and diseases, these discrepancies limit their ability to fully substitute human bone tissue.

### 2.5 Higher-order bone organoids: beyond osteogenesis

Bone serves as an essential component of the human body, providing structural support and facilitating hematopoiesis 30. The support function relies on the deposition of inorganic minerals within the bone matrix, primarily hydroxyapatite, which endows the bone with its mechanical strength and hardness 31. However, the functionality of bone extends beyond mere structural support, encompassing roles in hematopoiesis. The bone marrow harbors hematopoietic stem cells (HSCs) that differentiate into various blood cells, thus maintaining the body's circulatory and immune systems 32.

In the development of higher-order bone organoids, it is imperative to replicate not only the mechanical properties of bone but also its hematopoietic functions. Therefore, the definition of a high-fidelity bone organoid must consider several key aspects: it should effectively mimic the mineralization process of natural bone to form tissue with adequate mechanical strength; create a niche similar to the bone marrow to support the growth and differentiation of hematopoietic stem cells; facilitate the production and functionality of immune cells, as the bone marrow is pivotal for immune cell development; emulate the intricate interactions between the skeletal and nervous systems, where neural innervation significantly influences bone growth and repair; maintain immune homeostasis through the connectivity between the bone marrow and the lymphatic system; and ensure proper vascularization to provide adequate nutrient and oxygen supply for sustaining tissue growth and maintenance. The successful development of such bone organoids demands a multidisciplinary approach, integrating materials science, cell biology, immunology, and bioengineering to create complex, functionally comprehensive, and structurally sophisticated tissue-engineered products.

### 2.6 Developmental stages of bone organoids

The classification of bone organoids is generally based on their histological characteristics. Traditional classifications divide them into types such as osteo-callus organoids, woven bone organoids, trabecular bone organoids, bone marrow organoids, cartilage organoids, and dental pulp organoids. Despite the differing names, osteo-callus, woven bone, and trabecular bone organoids all simulate the morphology and mineralization processes of trabecular bone 33. Woven bone organoids emphasize the multidirectional differentiation potential of stem cells 34, while osteo-callus organoids focus on replicating the pathological state of fracture healing, particularly the hard callus during the remodeling phase, with the added capability of autonomously forming ectopic bone micro-organs 35. Bone marrow organoids, derived from iPSCs, mimic bone marrow tissue, featuring an *in vivo*-like vascular network that supports neutrophil differentiation, and are used to study hematopoietic development and bone marrow diseases 36, 37. Cartilage organoids are designed to model cartilage tissue 38, while dental pulp organoids are developed using odontogenic epithelial stem cells to simulate the physiological functions of tooth mineralization 39, 40.

In line with the development and key research areas of bone organoids, our team has further categorized them into physiological, pathological, structural, composite, and application-based stages, providing a more systematic approach for research and application 41, 42. Physiological bone organoids simulate bone tissue under normal conditions, providing basic functions for fundamental research. Pathological bone organoids replicate the disease-related features of bone disorders, serving as tools for disease mechanism studies and drug screening. Structural bone organoids aim to closely mimic the architecture and functionality of bone, supporting advancements in bone repair and regenerative medicine. Composite bone organoids integrate multiple tissues, such as joints, periosteum, tendons, nerves, and blood vessels, allowing for more comprehensive investigations. Applied bone organoids are designed for rapid and large-scale production to support clinical applications and personalized medicine.

**Expert consensus 1:** Current global research on bone organoids remains at a preliminary stage, with these organoids only replicating a small portion of bone tissue functions. Consequently, the definition of bone organoids should evolve accordingly. In addition to the conventional functionalities and key attributes of organoids, bone organoids should possess both micro and macrostructures of bone, provide adequate mechanical support, contain a bone marrow microenvironment with hematopoietic capabilities, generate immune cells, and establish effective connections with the nervous, immune, lymphatic, and vascular systems. These characteristics will enable bone organoids to more closely mimic the physiological functions and structures of natural bone, advancing research and applications in this field.

## 3. How to create bone organoids?

**Figure 3:**
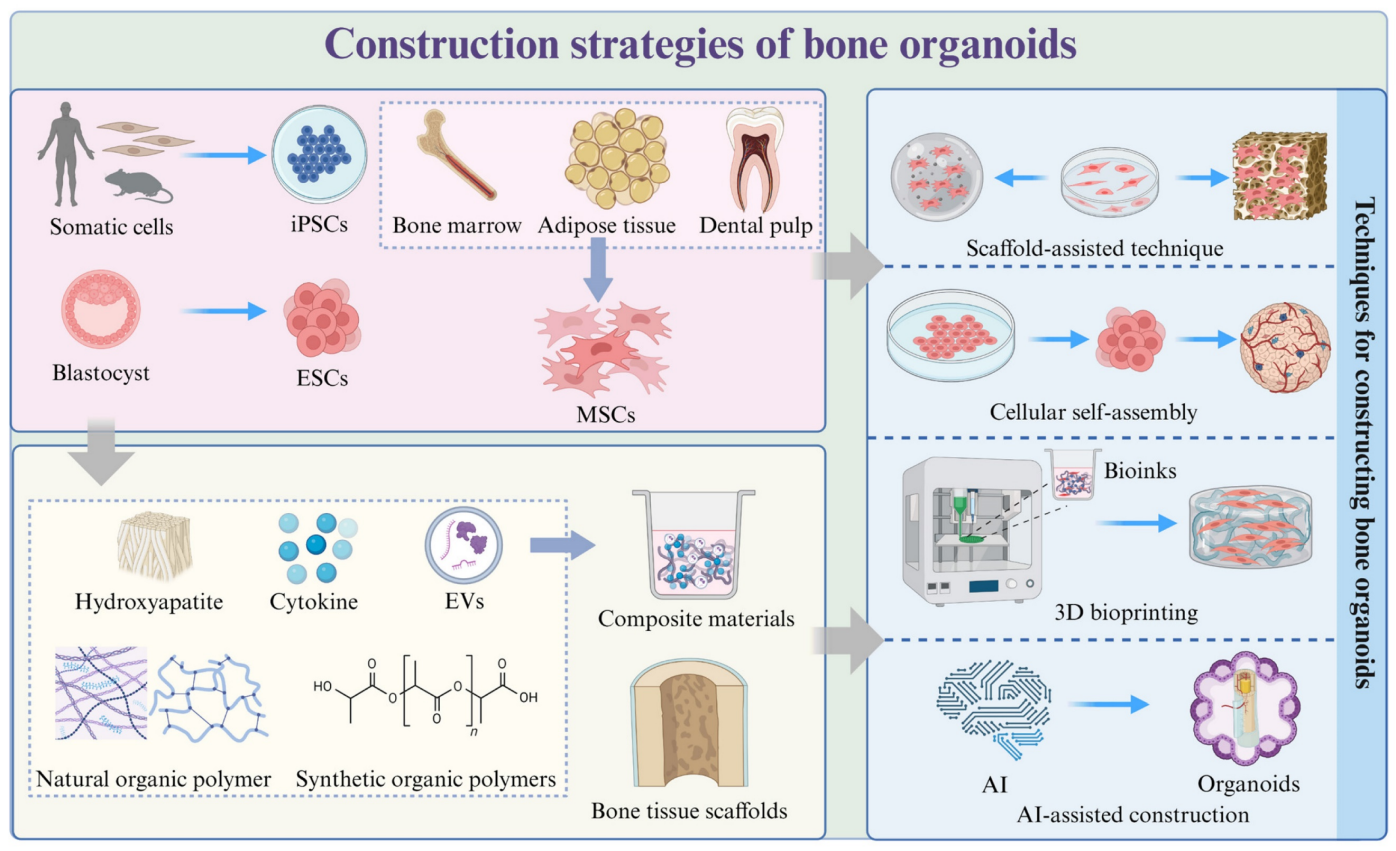
** Construction strategies of bone organoids.** Strategies for constructing bone organoids include using various stem cell sources, such as iPSCs derived from somatic cells, ESCs from blastocysts, and MSCs from bone marrow, adipose tissue, and dental pulp. These stem cells can be cultured and assembled into bone-like structures using techniques like scaffold-assisted approaches, cellular self-assembly, 3D bioprinting, and AI-assisted construction. Common materials used in the construction process include hydroxyapatite, cytokines, extracellular vesicles (EVs), natural and synthetic organic polymers, which are often combined to create composite materials for bone organoids.

Creating bone organoids involves a series of carefully controlled steps to mimic the complex environment of bone tissue **(Figure 3)**. This process requires a deep understanding of the biological, chemical, and physical factors that influence bone development and function 43. By recreating these conditions *in vitro*, scientists aim to produce organoids that not only resemble the architecture of natural bone but also exhibit similar functional properties. These steps include selecting the appropriate cell types, designing a suitable 3D culture environment, implementing differentiation protocols, and ensuring proper maturation and vascularization of the organoids 44. Through meticulous control and optimization of these processes, researchers can generate bone organoids that serve as powerful models for studying bone biology, disease mechanisms, and potential therapeutic interventions 45.

### 3.1 Selection of seed cells

The choice of starting cell population for any organoid significantly impacts the variability, heterogeneity, and functionality of the final structure 46 **(Table 1)**. Seed cells serve as the foundation for constructing bone organoids, requiring high proliferative capacity and multipotent differentiation potential, enabling them to differentiate into bone-related cells under suitable conditions 23.

#### 3.1.1 Pluripotent stem cells (PSCs)

Directed differentiation of pluripotent stem cells (PSCs), including ESCs and iPSCs, involves a step-by-step approach that replicates the natural transition from the epiblast stage to specific tissue types 47. This method is more efficient than single-step approaches and has been successfully employed to generate a variety of human tissues.

Currently, there are various established protocols for constructing organoids of the lung 48, cerebral 49, kidney 47, blood vessels 50, cochlea 51, heart 52, intestinal 53, and liver 54 using PSCs. iPSCs are a revolutionary type of stem cell generated by reprogramming adult somatic cells, such as skin or blood cells, to revert to a pluripotent state. This process involves the introduction of specific transcription factors that reset the cells' identity, granting them the remarkable ability to differentiate into any cell type found in the body 55. iPSCs are widely used as the most common seed cells for bone organoid construction due to their remarkable pluripotency and self-renewal capabilities. Guilak et al. developed an osteochondral organoid using murine iPSCs, successfully modeling the process of endochondral ossification 56. Psaila et al. induced iPSCs to differentiate into mesenchymal, endothelial, and hematopoietic lineage cells, which then self-assembled into 3D structures to mimic the complex microenvironment of human bone marrow 37. The construction process involved inducing iPSCs to form mesodermal aggregates, differentiating into vascular and hematopoietic cells, and ultimately producing mature bone marrow organoids under specific culture conditions. In another similar study, Klein et al. induced iPSCs to form non-adherent mesodermal aggregates, which were differentiated into mesenchymal, endothelial, and hematopoietic lineage cells, successfully generating mature bone marrow organoids containing these cell types 36.

#### 3.1.2 Mesenchymal stem cells (MSCs)

MSCs are a type of stem cell with multi-lineage differentiation potential, primarily sourced from bone marrow, adipose tissue, umbilical cord, and placenta 57. Under specific *in vitro* conditions, MSCs can differentiate into various bone-related cells, including osteoblasts, chondrocytes, and adipocytes. This differentiation capability makes MSCs ideal seed cells for constructing bone organoids. Ehrbar et al. used bone marrow-derived stromal cells and human hematopoietic stem and progenitor cells (HSPCs) to construct bone marrow organoids 58. By simulating the *in vivo* microenvironment *in vitro*, MSCs can be induced to differentiate into specific cell types, promoting the formation and maturation of bone organoids. Xing et al. used human umbilical cord MSCs to construct osteochondral organoids 59. Bone marrow mesenchymal stem cells (BMSCs) are multipotent stem cells found in the bone marrow. They can be cultured and expanded *in vitro* while retaining their differentiation potential and self-renewal capabilities, with the ability to differentiate into various types of bone tissue cells. Su et al. constructed bone organoids with self-mineralization and multicellular differentiation capabilities by combining BMSCs with bone matrix-inspired bioink 26. After implantation into nude mice, these bone organoids formed structures similar to natural bone tissue. Ouyang et al. used human and rabbit BMSCs to construct microspheres containing gelatin methacrylate (GelMA) 60. After chondrogenic induction and culture, these microspheres successfully transformed into osteo-callus organoids. Tampieri et al. constructed a 3D bone organoid using rat BMSCs and mouse embryonic fibroblasts (Swiss 3T3) 61. Dental pulp stem cells (DPSCs) are a type of mesenchymal stem cell found in the dental pulp of teeth. Bai et al. constructed prevascularized dental pulp organoids using human DPSCs 62. Similar to BMSCs and DPSCs, periosteal skeletal stem/progenitor cells (SSCs) also exhibit excellent properties in bone formation 63. During bone injury, mechanical stretching or small molecules can activate the differentiation of periosteal SSCs, rapidly generating osteoblasts to repair bone 64. Papantoniou et al. used human periosteum-derived cells to construct callus organoids 35. This suggests that stem cells derived from the periosteum may be ideal seed cells for constructing bone organoids. Adipose-derived stem cells (ASCs) are mesenchymal stem cells obtained from adipose tissue. They possess multilineage differentiation potential and excellent self-renewal capabilities, maintaining their stemness and differentiation ability during long-term *in vitro* culture, which supports bone tissue formation and mineralization 65. ASCs are abundant, easy to obtain, and cause minimal damage to donors. Their self-renewal and proliferative abilities, along with their immunomodulatory properties, help reduce post-transplant rejection 66. Therefore, ASCs are considered an ideal cell source for constructing human bone organoids in the future.

#### 3.1.3 Other types of somatic cells

In the study of bone organoids, various types of somatic cells are used as seed cells to simulate different physiological and pathological processes. Osteoblasts are employed to investigate bone formation and mineralization, while osteoclasts are involved in modeling bone resorption, facilitating the study of bone remodeling and diseases such as osteoporosis 67. A micron-scale bone organoid prototype was developed by seeding osteoblasts and osteoclasts onto femoral head micro-trabeculae, simulating pathological bone loss and enabling the study of mechanisms underlying bone remodeling imbalances 33. Fibroblasts participate in bone repair and regeneration, chondrocytes help to model the process of endochondral ossification, and vascular endothelial cells support microvascular formation in organoids, enhancing their physiological relevance. Bone marrow stromal cells can simulate the bone marrow environment, supporting bone development and disease research. Additionally, immune cells such as macrophages and T cells play significant roles in inflammation and bone remodeling, and their inclusion aids in studying the interactions between the immune system and bone tissue. Osteogenic cells and bone marrow mononuclear cells (BMMs) extracted from DsRed and GFP mice were used to develop trabecular bone organoids 68. The osteogenic cells were differentiated on demineralized bone paper (DBP), while BMMs were differentiated into osteoclasts in a specific culture medium. The co-culture of these cells was used to simulate the bone remodeling process. Cottrell et al. used a murine pre-osteoblast cell line (MC3T3-E1 cells) to differentiate into osteoblasts and a murine monocyte cell line (RAW 264.7) to differentiate into osteoclasts to construct 3D bone organoids 69. Metcalfe et al. used human osteoblasts and osteoclast precursors to construct bone organoids to analyze the effects of microgravity on bone remodeling and degeneration 33.

**Expert consensus 2:** For the construction of bone organoids, PSCs and MSCs are recommended. In specific disease models, stem cells and primary cells can be derived from patient tissues.

Incorporating cells from multiple sources, such as stem cells and adult cells, can further increase the complexity and functionality of bone organoids.

### 3.2 Matrix materials

In the process of constructing bone organoids, selecting appropriate matrix materials can effectively simulate the characteristics of the natural bone environment, thereby better supporting the formation and function of bone tissue 13 **(Table 2)**. These materials provide a 3D framework that supports cell proliferation, migration, and differentiation. By maintaining the stemness of cells and ensuring a steady supply of nutrients, the matrix materials facilitate healthy cell development 70. They also provide the necessary mechanical support, helping to maintain structural integrity and elasticity. Furthermore, these materials can actively induce mineralization and bone formation, making them indispensable for developing functional bone organoids 71. The matrix materials used in bone organoid construction can be broadly categorized into natural organic polymers, synthetic organic polymers, composite materials, and bone tissue scaffolds, each bringing unique properties and advantages to the tissue engineering process 72.

#### 3.2.1 Natural organic polymer

Natural organic polymers are derived from biological sources, making them highly compatible with living tissues 73. These materials are integral to tissue engineering as they mimic the extracellular matrix (ECM) of natural tissues, providing essential structural and biochemical support to surrounding cells 74. Natural organic polymers offer a supportive environment for cell attachment, proliferation, and differentiation, facilitating the development of functional bone organoids.

##### Matrigel

Matrigel is one of the most commonly used materials for constructing organoids, including bone organoids 14. It is derived from the basement membrane extract of Engelbreth-Holm-Swarm (EHS) mouse sarcoma, a tumor rich in ECM proteins. Matrigel contains a complex mixture of laminin, collagen IV, heparan sulfate proteoglycans, and growth factors, which closely mimic the natural ECM environment 75. Advantages of Matrigel include its ability to support 3D cell culture and differentiation by providing essential biochemical signals and structural support. Matrigel is highly versatile and can be used in various applications, including the development of bone organoids, where it helps to maintain the stemness of cells and promotes osteogenic differentiation 76. Coelho et al. obtained samples from pediatric patients' bone and cartilage tissues, digested them, and then cultured the cells in cold Matrigel to construct bone and cartilage organoids 77. Cottrell et al. constructed a 3D bone organoid model using murine MC3T3-E1 and RAW 264.7 cell lines embedded in Matrigel to study bone homeostasis and related diseases 69. In the process of constructing bone marrow organoids, researchers typically use Matrigel and collagen as culture matrices to induce and support the differentiation of iPSCs 27, 36, 37. These matrix materials provide a 3D environment that promotes the differentiation of iPSCs into specific types of bone marrow cells, thereby forming organoids. However, Matrigel has some limitations 78. As it is derived from a mouse tumor, it can introduce batch-to-batch variability and potential contamination with mouse proteins, which may affect reproducibility and translational applications. Additionally, Matrigel is not entirely defined, making it challenging to control its composition precisely, which may limit its use in certain clinical and regulatory settings.

##### Collagen

Collagen is a class of fibrous structural proteins composed of polypeptide chains, widely present in the skin, bones, tendons, ligaments, blood vessels, cornea, and connective tissues 79. In the construction of bone marrow organoids, researchers typically incorporate Type I and Type IV collagen to better mimic the natural microenvironment of the bone marrow 36, 37. Type I collagen, abundantly present in the body, provides structural support, particularly in bone and connective tissues. It mimics the extracellular matrix of bone tissue, facilitating the attachment and differentiation of osteoblasts and mesenchymal stem cells 79. Type IV collagen, a major component of the basement membrane, creates a microenvironment with specific biochemical signals that promote the attachment of endothelial cells and the formation of blood vessels, thereby simulating the microvascular system within the bone marrow 80. Additionally, Type IV collagen helps maintain the stability of the cellular microenvironment, working in conjunction with other matrix components such as laminin to support the functionality of hematopoietic and stromal cells 81. For example, a study found that the osteogenic organoid, composed of allogeneic mesenchymal stem cells combined with collagen microbeads and platelet-rich plasma clots, effectively induces bone formation and promotes lesion healing in patients with congenital pseudoarthrosis of the tibia 82. However, collagen has drawbacks such as low mechanical strength, uncontrollable degradation rates, potential immunogenicity, and limited plasticity, which is why it is often combined with other matrix materials to address these limitations and improve its overall effectiveness in applications.

##### Gelatin

Gelatin is widely used in the construction of bone organoids due to its biocompatibility, biodegradability, and ability to mimic the natural ECM 83. By crosslinking to form hydrogels, it provides a 3D scaffold that supports the development of bone-like tissue 84. Xing et al. utilized gelatin-based microcryogels to self-assemble into osteochondral organoids, achieving simultaneous regeneration of cartilage and bone 59. As a derivative of collagen, gelatin effectively simulates the natural ECM of bone tissue, promoting cellular interactions, osteogenic differentiation, and matrix deposition 85. Additionally, the degradation rate of gelatin hydrogels can be adjusted to match the pace of tissue development, thereby maintaining a balance between scaffold support and tissue maturation 86. To enhance the mineralization process, gelatin can also be combined with hydroxyapatite or bioactive peptides. Its customizability allows it to integrate with bioactive molecules or mechanical signals, creating a more responsive environment that better replicates the mechanical properties and biological functions of natural bone. Su et al. developed bioinks mimicking bone matrix by combining GelMA, alginate methacrylate (AlgMA), and hydroxyapatite, and used 3D bioprinting to construct bone organoids 36. In another study, Ouyang et al. constructed osteo-callus organoids by 3D bioprinting GelMA microspheres enriched with BMSCs, followed by *in vitro* culture 60. However, gelatin tends to lose its structure and mechanical properties at higher temperatures, and the introduction of photocrosslinking groups or crosslinkers may pose potential toxicity risks.

##### Alginate

Alginate is a natural polysaccharide extracted from brown algae, composed of mannuronic acid and guluronic acid units linked by covalent bonds 87. It has the ability to crosslink with divalent cations such as calcium ions, forming a highly hydrated gel. This gel-forming property makes alginate widely used in bone tissue engineering, particularly in bioprinting as a cell support material and delivery system 26. Its excellent biocompatibility, non-toxicity, and biodegradability allow it to provide a soft and elastic 3D environment for cells, supporting their survival, proliferation, and differentiation. Alginate hydrogels effectively promote the survival and development of human intestinal organoids through mechanical support, with differentiation outcomes comparable to organoids cultured in Matrigel 88. Hui et al. constructed liver organoids by encapsulating human hepatocytes with alginate, effectively improving liver failure in animal models 89. Additionally, the mechanical strength and degradation rate of alginate can be adjusted according to specific application needs, better simulating the physiological environment in the body. Mooney et al. encapsulated kidney organoids in viscoelastic alginate hydrogels with varying rates of stress relaxation to investigate the effects of mechanical signals on nephron patterning and morphology in 3D culture 90. Ito et al. encapsulated rapidly self-assembling cartilage organoids in alginate hydrogels with varying viscoelastic properties, discovering that viscoelastic hydrogels promoted the fusion of organoids into neocartilage rich in collagen type II and glycosaminoglycans 91. Alginate promotes organoid generation and effectively maintains their normal phenotypic structure. Proteomic analysis of long-term cultured kidney organoids revealed that excessive accumulation of collagen types 1a1, 2, and 6a1 is a hallmark of renal fibrosis. Encapsulating kidney organoids in a soft, thiol-ene cross-linked alginate hydrogel successfully reduced the abnormal expression of collagen type 1a1, laying the foundation for their clinical application 92. For example, alginate hydrogels can replace Matrigel in spinal cord organoid generation, reducing organoid size variability while effectively supporting neurogenesis and gliogenesis, and demonstrating superior control over neural fate specification 93. Due to the lack of cell recognition sites in alginate, its poor cell adhesion affects cell growth, proliferation, and differentiation. Therefore, it is often necessary to combine it with bioactive materials such as collagen or hydroxyapatite to promote bone tissue formation and regeneration.

##### Hyaluronic acid

Hyaluronic acid, a polysaccharide naturally found in connective tissue and synovial fluid, is highly valued for its exceptional water retention capacity and biocompatibility, making it an ideal component in organoid matrices 94. It provides the necessary hydrated environment and mechanical support to maintain cell viability while promoting cell proliferation and migration. For example, the hyaluronic acid hydrogel scaffold can significantly enhance the maturation of self-assembling organoids and promote their functional differentiation 95. Furthermore, hyaluronic acid interacts with cell surface receptors to effectively regulate the proliferation and differentiation of osteoblasts, thereby facilitating bone tissue formation and functional recovery 96. Hyaluronic acid combines with other materials to form composite hydrogels, providing an ideal matrix for the construction of bone organoids that more closely mimics the physiological environment. Ehrbar et al. encapsulated stem cells in hybrid hydrogels made of polyethylene glycol (PEG) and hyaluronic acid, utilizing PEG for mechanical stability and Hyaluronic acid for enhancing biological interactions, to construct physiologically relevant bone marrow organoid models 58.

##### Chitosan

Chitosan is a natural polysaccharide extracted from the shells of crustaceans, known for its excellent antibacterial properties, biodegradability, and biocompatibility 97. As a scaffold material, chitosan effectively supports cell adhesion, proliferation, and differentiation, while gradually degrading in the body, eliminating the need for secondary surgeries 98. Its antibacterial properties are particularly beneficial for preventing infections around implants, ensuring the safety and functionality of organoids. Shao et al. successfully induced the formation of spinal cord organoids from PSCs by engineering composite microspheres made of porous chitosan microspheres combined with Matrigel 99. In drug delivery, the cationic nature of chitosan makes it an ideal carrier, capable of precisely delivering growth factors, extracellular vesicles (EVs), or other bioactive molecules within bone organoids, thereby promoting their growth and development 100.

##### Cellulose and its derivatives

Cellulose is a primary component of plant cell walls, composed of glucose units linked by β-1,4-glycosidic bonds 101. Cellulose, due to its structural stability, wide availability, and immune compatibility, is used as a scaffold material to provide physical support in organoid construction. Research by Spee et al. demonstrated that cellulose nanofibril hydrogel can serve as a clinical-grade scaffold alternative to Matrigel for liver organoid differentiation, with superior mechanical properties and cell functionality compared to Matrigel 102. In another study, Garnier et al. found that hydrogels formed by combining cellulose nanofibers with type I collagen serve as a thermo-responsive matrix that supports the formation and growth of intestinal organoids, with superior mechanical properties and cell activity compared to traditional animal-based matrices 103. Additionally, cellulose derivatives, such as methylcellulose and carboxymethylcellulose, play an even more significant role in organoid construction 104. Methylcellulose, by partially substituting the hydroxyl groups in cellulose molecules, enhances solubility and viscoelasticity, exhibiting excellent rheological properties in hydrogels and bioinks, making it particularly suitable for 3D bioprinting to precisely construct the complex structures of organoids 105. Carboxymethylcellulose, through the introduction of carboxymethyl groups, improves the material's water solubility and bioactivity, forming stable gels under physiological conditions, providing a more ideal microenvironment for organoid generation 106. Although research on the use of cellulose materials in the construction of bone organoids is currently limited, their sustainability, customizability, and cost-effectiveness highlight their potential as valuable support materials in future bone tissue engineering.

##### Silk fibroin

Silk fibroin is a natural protein extracted from silk, rich in β-sheet structures that provide it with high strength and elasticity, while also allowing it to gradually degrade under physiological conditions, producing non-toxic byproducts 107. As a scaffold material, silk fibroin not only offers robust physical support for cells but also promotes cell adhesion and proliferation through its unique structural properties, aiding in bone tissue repair and regeneration 108, 109. Its versatility in processing allows silk fibroin to be fabricated into various forms, such as porous structures, films, or fibers, using methods like electrospinning, freeze-drying, or 3D printing, making it adaptable to different tissue engineering applications. Su et al. utilized silk fibroin and DNA hydrogels to construct microspheres, significantly enhancing the chondrogenic differentiation of BMSCs, offering an innovative material choice and strategy for the construction of cartilage organoids 110.

##### Decellularized extracellular matrix (dECM)

dECM provides a biologically active scaffold for organoid construction by removing cellular components from natural tissues while retaining key structural proteins and bioactive molecules such as glycosaminoglycans 111. dECM can be derived from a wide range of tissues, including the heart, liver, and skeletal muscle. After decellularization, the preserved bioactive molecules not only offer physical support to cells but also regulate cellular behaviors such as proliferation, migration, and differentiation, thereby maintaining the matrix's functional properties 112. By mimicking the *in vivo* microenvironment, dECM promotes the self-organization and growth of organoids. Furthermore, the source of dECM can be tailored to specific needs, with liver-derived dECM supporting liver organoid formation and bone-derived dECM facilitating the mineralization and remodeling of bone organoids 113-115. This versatility enhances the potential applications of organoid technology.

#### 3.2.2 Synthetic organic polymers

Synthetic organic polymers offer a versatile platform for constructing bone organoids by mimicking the ECM and supporting cell growth. Their tunable properties enhance stem cell differentiation into osteogenic lineages, promoting bone tissue formation *in vitro*.

##### Polyethylene glycol (PEG)

PEG, as a highly biocompatible synthetic polymer, has shown broad application potential in organoid construction 116. Through chemical modifications, PEG can regulate its mechanical properties and degradation rate, providing an ideal 3D scaffold for organoid growth 117. With its high water content and excellent permeability in hydrogel form, PEG mimics the extracellular matrix environment* in vivo*, promoting cell growth, differentiation, and organization within organoids, thereby supporting the formation of complex tissue structures 118. For example, humanized bone marrow organoid models were successfully constructed by encapsulating stem cells in PEG-based hydrogels, utilizing the mechanical stability of PEG 58.

##### Poly (lactic-co-glycolic acid) (PLGA)

Poly (lactic-co-glycolic acid) (PLGA) is a biodegradable synthetic polymer composed of lactic acid and glycolic acid monomers, widely used in drug delivery, tissue engineering, and organoid construction. In organoid research, PLGA is commonly utilized to fabricate 3D scaffolds or microcarriers, providing necessary structural support while promoting cell adhesion, proliferation, and differentiation, thereby facilitating the functionalization and maturation of organoids. By adjusting the degradation rate of PLGA, its application can be synchronized with organoid development, further optimizing its effectiveness. For example, Chen et al. utilized PLGA loaded with cell-free fat extract (Ceffe) to construct a porous nipple-shaped scaffold, which could continuously release Ceffe and promote cartilage organoid formation 119.

##### Polylevolactic acid (PLLA)

Poly-L-lactic acid (PLLA) is a synthetic biodegradable polymer and a type of lactic acid polymer. As a scaffold material for constructing organoids, PLLA is gaining increasing attention due to its excellent biocompatibility and controllable degradation properties. The structural integrity and mechanical strength of PLLA provide a stable environment for the growth and development of organoids, while its degradability ensures that the scaffold gradually integrates with the surrounding tissue as the organoid matures. PLLA, as a scaffold for organoid seeding, can effectively promote the formation of organoids and develop into tissue similar to human tissue 120. By utilizing PLLA, researchers can create well-defined 3D environments to support the formation and functionality of various organoids. For example, a 3D bone organoid was constructed by using PLLA to reconstruct vascular structures and mimic the microarchitecture of flat and short bones, incorporating BMSCs and Swiss 3T3 cells on a collagen-PLLA composite 61.

#### 3.2.3 Composite materials

Single matrix materials are insufficient to meet the complex requirements of bone matrices in bone organoid construction 13. To address this challenge, researchers have developed composite materials that combine the mechanical properties and bioactivity of different components, thereby better mimicking the microenvironment and biological functions of bone tissue 121. These composite materials provide the necessary support for cell adhesion, proliferation, and differentiation, facilitating the construction of functional bone organoids. For example, hydrogels combined with inorganic nanoparticles can simulate the mineralized environment of bone tissue, promoting osteoblast differentiation and mineralization 26. Polymer-ceramic composites combine osteoconductivity and mechanical support, aiding in bone repair and regeneration 122. Natural-synthetic composites offer both bioactivity and tunable mechanical properties, making them suitable for bone organoid growth 58. Additionally, smart responsive materials can adapt their properties in response to external stimuli, mimicking the dynamic environment of bone tissue and further promoting the functional maturation of organoids 123.

#### 3.2.4 Bone tissue scaffolds

Natural bone tissue scaffolds, used as matrix materials for bone organoids, are typically composed of decellularized bone tissue and bone matrix proteins. These scaffolds exhibit good biocompatibility and bioactivity, facilitating cell adhesion, proliferation, and differentiation. Retaining the microstructure of bone tissue, they provide physical and mechanical support similar to the *in vivo* environment, thereby promoting bone cell function and regeneration. For example, Iordachescu et al. developed a micron-scale bone organoid prototype by seeding primary osteoblasts and osteoclasts onto femoral head micro-trabeculae to simulate pathological bone loss and study the cell-tissue interface mechanisms underlying bone remodeling imbalances 33. To ensure their suitability for tissue engineering and regenerative medicine, natural bone tissue scaffolds usually undergo decalcification and decellularization processes. Decalcification involves the use of acidic solutions to remove inorganic mineral components while preserving organic elements such as collagen, thereby softening the scaffold and enhancing cell adhesion. Decellularization employs detergents, enzymes, or mechanical methods to remove cellular components, retaining the 3D structure of the ECM and reducing the risk of immune rejection. Toni et al. constructed a bioartificial 3D bone organoid based on a decellularized and decalcified matrix, successfully replicating the microanatomy of flat and short bones in mammals 61.

**Expert consensus 3:** The use of both organic and inorganic materials is recommended for constructing bone organoids. Organic materials, such as collagen and Matrigel, offer biocompatibility and cellular support, while inorganic materials, like hydroxyapatite and calcium phosphate, improve mineralization and mechanical properties. Future research should prioritize the development of more advanced matrix materials to address the limitations of those currently available.

### 3.3 Construction methods

The construction strategies for bone organoids involve the integration of various engineering approaches and biological techniques, aiming to closely mimic the structure and function of *in vivo* bone tissue to meet the demands of clinical applications and basic research.

#### 3.3.1 Scaffold-assisted technique

The cell-scaffold composite technology simulates the structure and function of bone tissue by integrating cells with biomaterial scaffolds. The selection of appropriate scaffold materials requires consideration of mechanical properties, pore structure, biocompatibility, and degradation characteristics. Luyten et al. differentiated iPSCs into chondrocytes, embedding them in a 3D scaffold for the repair of long bone defects 124. Imazato et al. utilized DPSCs and BMSCs to construct vascularized bone organoids, creating cell aggregates expressing the endothelial marker CD31, which significantly improved cell viability and promoted matrix mineralization 125. During cell seeding, careful optimization of density and method ensures uniform distribution and maximizes cell viability. Common techniques include dynamic rotary or static culture methods for seeding osteoblasts or stem cells onto the scaffold. The attachment, proliferation, and differentiation of cells on the scaffold are key factors. By regulating the chemical properties and physical morphology of the scaffold surface, cell adhesion and proliferation rates can be enhanced. Surface modification or nano-coating techniques can improve the biocompatibility of scaffold materials, promoting cell proliferation and differentiation. Xing et al. developed gelatin-based microcryogels, modified with hyaluronic acid and hydroxyapatite, to induce cartilage and bone regeneration, forming a seamless biphasic cartilage-bone organoid *in vivo* 59. By injecting bioink containing BMSCs, ECM, and growth factors into bone defects, the natural assembly and cell-cell interactions within the* in vivo* microenvironment are leveraged to promote bone tissue regeneration and repair.

#### 3.3.2 Cellular self-assembly

The basic principle of cellular self-assembly technology involves the biological acquisition of cells and the precise regulation of their morphology, type, quantity, and distribution, enabling cells to self-organize into the desired structures 126. By leveraging the innate self-assembly capability of cells, complex 3D tissues can be formed through specific culture conditions and signaling induction 127. The core of this technology lies in selecting appropriate cell types, such as stem cells and progenitor cells, and optimizing culture conditions to promote cellular self-assembly and differentiation. Through the use of growth factors and matrix proteins as inductive agents, the spatial arrangement and interactions of cells within the 3D space are precisely controlled. Additionally, external stimuli such as mechanical stress and electrical signals are modulated to guide the formation of the intended tissue structures and functions 128. One study reported the development of complex vascularized bone marrow organoids by culturing iPSCs in a mixed collagen and Matrigel hydrogel, with phased addition of growth factors and optimized culture conditions 37. In another study, researchers directed the differentiation of iPSCs, utilizing cytokines and Matrigel hydrogels to self-assemble into 3D multi-lineage bone marrow organoids within 18 days 36. These organoids, comprising BMSCs, fibroblasts, endothelial cells, and hematopoietic cells, successfully mimicked the structure and function of natural bone marrow tissue. Papantoniou et al. developed a self-assembly method based on human periosteum-derived cells, where these cells spontaneously aggregate to form uniform microspheroids through scaffold-free microspheroid culture, and subsequently differentiate into callus organoids 35. Cellular self-assembly technology enables the formation of complex 3D tissues by selecting appropriate cell types, optimizing culture conditions, and integrating growth factors and external stimuli, effectively achieving the construction of intricate structures in bone organoid development.

#### 3.3.3 3D bioprinting

3D bioprinting technology enables the construction of complex 3D tissue structures by precisely controlling the layer-by-layer deposition of cells, matrix materials, and bioactive factors. Its advantages lie in its high precision and reproducibility, which meet clinical demands. The selection of bioinks directly affects the stability of the printing process and the functionality of the generated tissues, as their composition and physical properties, such as viscosity, biocompatibility, and mechanical strength, influence the quality and biological function of the constructed tissues 129. Optimizing parameters such as printing speed, temperature, and layer thickness directly influences the success of the printing process. Precise control of these parameters ensures high cell viability, uniform distribution, and promotes tissue formation and functional recovery 130. In a study, digital light processing (DLP) 3D printing technology was employed to fabricate GelMA microspheres containing BMSCs 60. Through chondrogenic induction and osteogenic differentiation, highly efficient callus organoids for bone regeneration were successfully developed. In another study, researchers utilized 3D bioprinting technology with a mixed bioink of GelMA, AlgMA, and hydroxyapatite to create self-mineralizing large-scale bone organoids 26. This method mimics the complexity of the ECM and constructs bone organoids with functionality and mechanical properties closely resembling natural bone tissue. Studies indicate that bioprinting technology significantly enhances the construction of bone organoids by precisely controlling the spatial distribution of cells and biomaterials, combining multiple biomaterials, and utilizing dynamic culture environments 131, 132.

#### 3.3.4 Artificial Intelligence (AI)

AI-driven organoid technologies are accelerating the progress of organoid research 133. In the construction of bone organoids, AI technologies enable precise modeling, optimization of culture conditions, and monitoring of growth, significantly improving experimental efficiency and accuracy 134. Through data-driven design, material optimization, printing parameter refinement, cell behavior prediction, dynamic culture systems, quality control, and personalized medical applications, AI substantially enhances the efficiency and precision of design, optimization, and manufacturing processes 131. AI can process large volumes of biomedical data to generate high-precision bone structure models and screen and optimize biomaterials suitable for bone organoid construction via 3D bioprinting. Additionally, AI can predict cell behavior under various culture conditions, optimizing medium composition and environmental factors to promote the formation and maturation of bone organoids 135. By monitoring and adjusting the physical and chemical conditions in dynamic culture systems in real time, AI ensures that cells grow and differentiate under optimal conditions, resulting in accurately structured and functional bone organoids. AI is also used for real-time detection and analysis of various parameters during the printing process, enabling the timely identification and correction of deviations. By integrating patient medical data, AI can customize personalized bone organoids for the repair of bone defects, thereby avoiding immune rejection and improving therapeutic outcomes.

### 3.4 Physical and biochemical cues

Physical and biochemical cues refer to the physical and biochemical signals used to guide cell behavior and tissue formation during organoid culture. These cues work synergistically to help simulate the *in vivo* microenvironment and facilitate successful organoid cultivation. Bone organoid formation and development require mimicking the bone microenvironment. Tissue mineralization is a key feature of bone organoids and is critical for their structure and function. Osteogenic mineralization in bone organoids requires modulation of calcium and phosphate concentrations in the culture medium. Calcium and phosphate ions aggregate to form mineralization cores, which ultimately lead to the deposition of mineralized hydroxyapatite crystals in the ECM. For example, introducing hydroxyapatite (HAP) nanoparticles into MSCs spheroids promotes their osteogenic differentiation into bone microtissues and their self-organization into bone organoids with a trabecular structure 136. Bone organoids are hard tissues under physiological conditions, and appropriate mechanical stimulation during culture is essential for their structural formation and functional maintenance, such as tensile or compressive stress. Mechanical stimulation can be achieved through dynamic culture systems or physical devices, such as microfluidic devices and *in vitro* bioreactors 137. Long-term mechanical loading significantly enhanced the mineral density, stiffness, osteoblast differentiation, collagen I maturation, and lacunar-canalicular network formation in bone organoids 132. Iordachescu et al. cultured trabecular bone organoids under simulated microgravity conditions and found that osteoclasts exhibited bone resorption patterns different from static controls 33. This organoid model simulates the physiological processes at the cell-tissue interface, revealing the mechanisms of pathological bone loss and imbalances in bone remodeling. Bone formation is regulated by various biochemical signals that guide cell differentiation, regulate tissue development, and promote bone mineralization 138. The use of stage-specific induction factors at different time points directs organoid cells toward specific tissue types. For example, Bethan Psaila's team constructed bone marrow organoids by adding specific biochemical factors at different time points, such as bone morphogenetic protein 4 (BMP4), fibroblast growth factor (FGF2), vascular endothelial growth factor A (VEGFA), stem cell factor (SCF), FMS-like tyrosine kinase 3 (FLT3), interleukin-3 (IL3), interleukin-6 (IL6), granulocyte colony-stimulating factor (G-CSF), erythropoietin (EPO), and thrombopoietin (TPO) 37. This process begins with the generation of iPSC aggregates, followed by stem cell induction, vascular formation, and hematopoietic cell differentiation, ultimately resulting in the formation of a 3D structure with bone marrow characteristics. By precisely controlling these physical and biochemical cues, the bone marrow microenvironment can be simulated, promoting the development and maturation of bone organoids. Additionally, it is necessary to establish a stable bone organoid culture system to ensure consistency and reproducibility of the culture conditions, which will facilitate more effective use of bone organoids in scientific research and clinical applications.

**Expert consensus 4:** The application of AI is recommended to optimize culture conditions, material design, and printing parameters, allowing for precise control of bone organoid structures through 3D bioprinting technology. Bone organoids with simple structural functions can be fabricated using cell-scaffold composite techniques or self-assembly approaches.

## 4. Why construct bone organoids?

**Figure 4:**
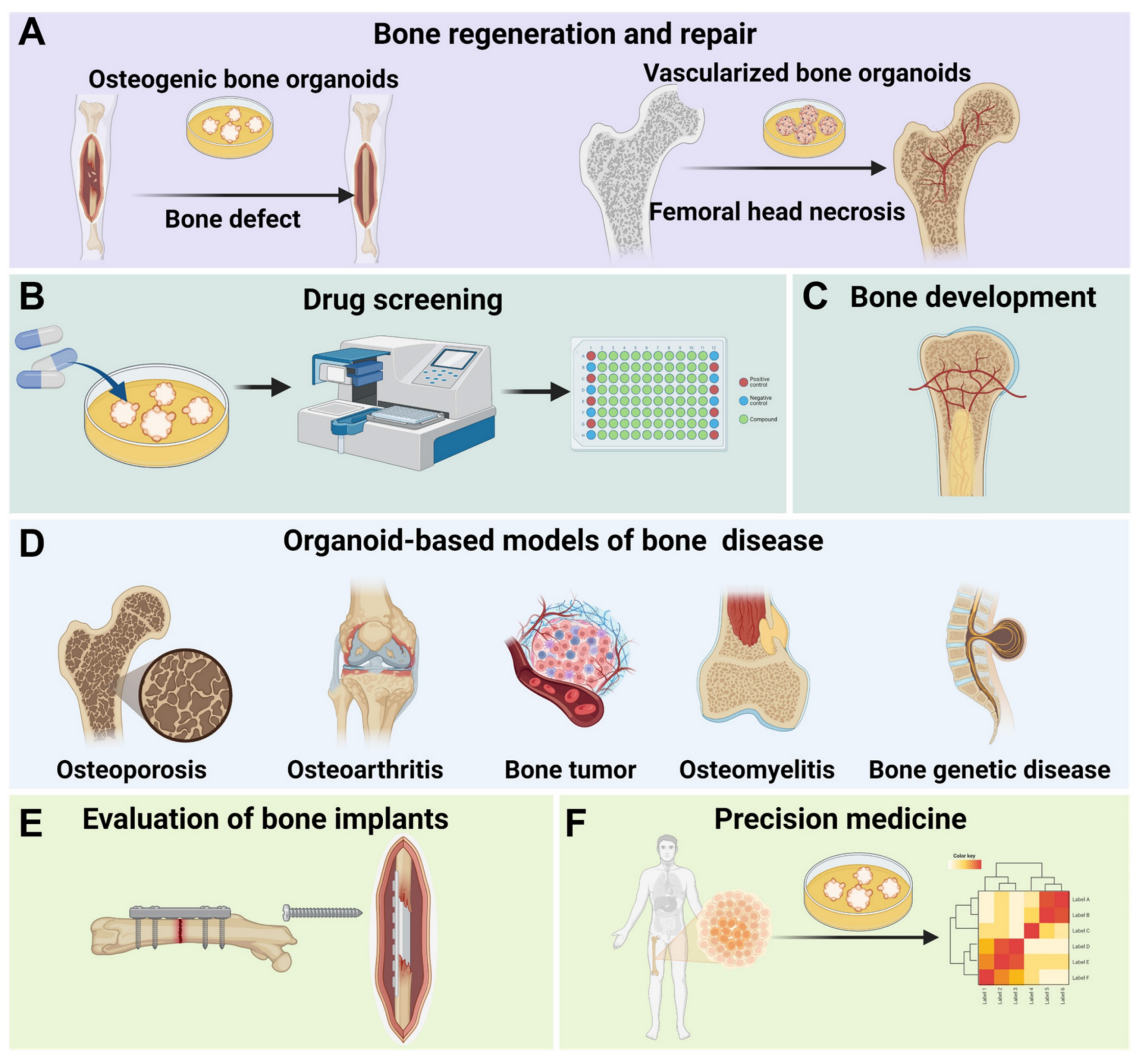
** Applications of bone organoids. (A)** Bone organoids are used for bone regeneration and repair, including the development of osteogenic and vascularized bone organoids for addressing bone defects and femoral head necrosis.** (B)** Bone organoids facilitate drug screening, allowing for the testing of compounds in a controlled environment. **(C)** Bone organoids are valuable in studying bone development processes. **(D)** Bone organoid-based models provide insights into various bone diseases, such as osteoporosis, osteoarthritis, bone tumors, osteomyelitis, and genetic bone diseases. **(E)** Bone organoids can be used to evaluate bone implants, assessing their compatibility and performance.** (F)** Bone organoids hold potential in precision medicine by enabling personalized treatment approaches based on patient-specific cellular data.

The construction of bone organoids can deepen our understanding of bone biology and improve the treatment of bone diseases 29. These organoids offer a more accurate, human-relevant model that can simulate the complexity and functionality of bone tissue, overcoming the limitations of traditional models like 2D cell cultures and animal studies 128. Bone organoids enable the study of bone development, disease mechanisms, drug responses, implant evaluations, and personalized medicine in a controlled environment, providing valuable insights for developing new therapies and addressing challenges in bone tissue engineering **(Figure 4)**.

### 4.1 Bone regeneration and repair

Bone regeneration and repair represent the most direct application of bone organoids 139. Traditional bone grafting methods face challenges such as donor shortages, immune rejection, and surgical risks, while tissue-engineered bone organoids offer significant advantages 140, 141. Bone organoids fabricated through 3D bioprinting and 3D culture techniques can precisely match the morphology and structure of a patient's bone defects, enabling personalized treatment 142. Moreover, large-scale bone defect repair encounters issues like insufficient vascularization, inadequate mechanical strength, and prolonged healing time. Bone organoids integrated with biomaterials and active cells can enhance graft functionality and promote bone defect healing 143. In the treatment of bone necrosis, vascularized bone organoids can be developed as therapeutic constructs to repair necrotic regions. These vascularized bone constructs mimic the 3D structure of natural bone tissue along with its intrinsic vascular network. By angiogenesis and enhancing osteogenic regeneration, vascularized bone organoids enhance local blood perfusion, modulate inflammatory responses, and provide essential mechanical support, thereby preventing further structural collapse of the necrotic bone region. This regenerative strategy presents a compelling and targeted approach to overcoming the challenges inherent in the treatment of ischemic bone necrosis.

**Expert consensus 5:** Bone regeneration and repair are the most immediate applications of bone organoids. To accelerate the translation of research into practical outcomes, it is essential to focus on constructing humanized bone organoids and establishing clear clinical translation pathways. These should include well-defined clinical trial designs, adherence to regulatory requirements, and effective market promotion strategies to facilitate the successful application of bone organoid technologies in clinical settings.

### 4.2 Drug screening

Bone organoids have significant applications in drug screening. Traditional drug screening relies on *in vitro* cell experiments and animal models, which often result in long development cycles and unpredictable long-term toxicity 144. As an innovative model, bone organoids simulate the *in vivo* microenvironment, providing realistic drug response data, reducing reliance on animal models, accelerating research and development, and lowering costs 7. For instance, the addition of A2A adenosine receptor agonists to bone and cartilage-derived organoids yielded experimental results consistent with those from cell and animal studies, validating the effectiveness of bone organoids as a drug testing platform 77. Although bone organoids are not yet widely used in drug screening for bone diseases, tumor organoids have demonstrated significant advantages in anticancer drug screening. Lung cancer organoids, for example, simulate the tumor microenvironment and are used in high-throughput drug screening and personalized treatment 145, 146. This suggests that bone organoids also hold potential for drug screening applications.

**Expert consensus 6:** The development of appropriate bone organoid disease models, along with high-throughput screening platforms, allows for the efficient evaluation of drug bioeffects by monitoring key parameters such as cell proliferation, differentiation, apoptosis, and mineralization. Furthermore, genomic and proteomic analyses should be applied to elucidate drug mechanisms, identify potential therapeutic targets, and facilitate personalized treatment approaches using patient-specific bone organoids.

### 4.3 Bone mechanism research

Organoids represent an innovative platform in biomedical research, providing a valuable means to study various physiological and pathological processes by replicating the 3D structure and function of human tissues. Bone organoids, in particular, offer the significant benefit of closely mimicking the physiological and pathological traits of bone tissue.

#### 4.3.1 Mechanisms of bone development

Bone development is a complex and dynamic process that includes the formation of the skeleton during embryogenesis through endochondral and intramembranous ossification. It involves the proliferation and differentiation of bone cells, mineral deposition, bone matrix remodeling, and vascularization, closely linked to the hematopoietic function of the bone marrow 147. In bone development research, the application of organoids is particularly important. Bone organoids can replicate the processes of bone tissue formation, mineralization, and maturation *in vitro*. While traditional 2D cell cultures and animal models have contributed to our understanding of basic bone development mechanisms, they fail to fully simulate the complex 3D environment and cellular interactions within the human body. Through organoid technology, researchers can construct highly biomimetic bone tissue models in the lab, allowing for more precise observation of bone cell behavior, matrix formation, and vascularization. The bone marrow, as the primary site of hematopoiesis, is embedded within the bone marrow cavity and is responsible for generating and releasing red blood cells, white blood cells, and platelets, maintaining proper blood circulation and immune function 148. By constructing 3D organoid models that incorporate the bone marrow microenvironment, researchers can more accurately simulate the *in vivo* hematopoietic process 36. These organoids not only replicate the proliferation, differentiation, and migration of hematopoietic stem cells in the bone marrow but also enable the study of the interactions between bone tissue and the hematopoietic microenvironment.

#### 4.3.2 Mechanisms of bone diseases

By constructing pathological models of bone organoids that replicate the molecular and cellular characteristics of diseases, researchers can gain a better understanding of the development of bone diseases and identify potential therapeutic targets, thereby enhancing the translatability and clinical relevance of experimental results 149, 150.

##### Organoid-based models of osteoporosis

Osteoporosis is the most common skeletal disease, characterized by bone resorption exceeding bone formation, leading to decreased bone density and strength, and increased bone fragility 151. Traditional animal models for osteoporosis are time-consuming and expensive to establish. Constructing osteoporosis models using organoids can shorten experimental cycles and reduce costs. By using collagen as a base material to simulate *in vivo* mineralization, a matrix microenvironment similar to normal bone tissue can be created. Introducing BMSCs and osteoclast precursors allows for the induced differentiation of osteoblasts and osteoclasts, controlling cell differentiation to regulate bone metabolism and simulate the imbalanced bone metabolism seen in osteoporosis. This approach offers new ideas for constructing osteoporosis-type bone organoids 68.

##### Organoid-based models of osteoarthritis (OA)

OA is a complex degenerative joint disease, with a pathophysiological process involving cartilage degeneration, subchondral bone proliferation, synovitis, and changes in joint fluid. Organoid-based OA models simulate the 3D structure and function of joint tissues, providing a new platform for studying the pathological mechanisms and therapeutic strategies of OA 152. Initially, pluripotent stem cells or primary cells are cultured until they reach a suitable state for differentiation. Then, using 3D bioprinting technology or self-assembly methods, organoids with dual bone and cartilage tissue structures are constructed to faithfully recreate the natural interface of the joint. During culture, by adding pro-inflammatory factors (such as IL-1β, TNF-α) or other pathological stimuli, pathological changes such as cartilage degeneration, subchondral bone proliferation, and synovitis can be induced 7, 153, 154.

##### Organoid-based models of bone tumor

Bone tumors are malignant or benign tumors that occur in the bones or associated tissues 155. Traditional tumor models lack the 3D structure, microenvironment, and cellular heterogeneity, leading to inaccurate drug responses and difficulties in studying tumor progression dynamically. Organoid-based bone tumor models have the advantages of simulating the 3D structure and function of the original tissue, supporting personalized medicine, and being suitable for long-term culture and high-throughput screening 156. These models allow dynamic studies of tumor development and reduce reliance on animal experiments. By isolating bone tumor cells from patients and constructing personalized bone tumor organoid models, researchers can use them for drug screening and personalized treatment 157. Rowley et al. explored the reactive stromal responses in the bone endosteum associated with prostate cancer metastasis to trabecular bone by co-culturing the prostate bone metastatic cell line (VCaP) with 3D osteogenic organoids 158. However, due to the complexity of bone tumor organoid structures, they have not yet been widely applied in drug screening. In contrast, liver and breast tumor organoids are already maturely used in personalized screening 159, 160. Deng et al. developed patient-derived breast cancer organoids that accurately simulate the histopathology and genetic characteristics of the original tumors, providing important references for personalized treatment plans by predicting drug sensitivity and reflecting previous treatment responses, significantly improving clinical outcomes 160.

##### Organoid-based models of osteomyelitis

Osteomyelitis is an inflammation of bone tissue caused by infection, usually triggered by the invasion of bacteria, fungi, or other microorganisms into the bone marrow cavity 161. Currently, treating osteomyelitis presents multiple challenges. By introducing infectious pathogens into bone organoids, researchers can simulate the pathological microenvironment of osteomyelitis, providing a highly physiologically relevant platform for studying the disease's pathogenesis and treatment options. Using cells extracted from patients or healthy donors, 3D *in vitro* culture techniques can construct osteomyelitis organoids that faithfully replicate the interactions between cells, inflammatory responses, and pathological features of osteomyelitis 7.

##### Organoid-based models of bone genetic disease

Organoid-based models of bone genetic diseases represent a cutting-edge research method. By combining patient or donor stem cells with gene editing technologies like CRISPR-Cas9, researchers can construct models of bone genetic diseases with specific gene defects 162. These models can accurately simulate the structure and function of bone tissue, including cell differentiation, mineralization, and metabolic activities, and can be used to study the pathological features of bone genetic diseases such as reduced bone density, structural abnormalities, and cellular dysfunction. They also allow for the evaluation of personalized treatment options. For example, the study of mutations in the *COL1A1* or *COL1A2* genes in osteogenesis imperfecta and mutations in the *TCIRG1* or *CLCN7* genes in osteopetrosis can help explore the potential applications of gene therapy and small molecule drugs in treating these conditions 163, 164. Another study showed that using human iPSCs expressing the *TNFRSF11B* gene readthrough mutation to construct cartilage and bone organoids revealed the role of osteoprotegerin with additional 19 amino acids at its C-terminus (OPG-XL) in subchondral bone turnover and cartilage mineralization 165.

**Expert consensus 7:** Gene editing and single-cell sequencing technologies provide valuable insights into gene expression profiles across various developmental stages of bone organoids. Real-time monitoring of organoid growth and development is made possible through the advancement of dynamic tracking technologies. Furthermore, the integration of big data and AI enables the identification of key regulatory factors. Promoting interdisciplinary collaboration will facilitate the adoption of innovative methods and technologies in bone organoid research.

### 4.4 Evaluation of bone implants

The preclinical evaluation of bone implant materials (such as medical metals, medical ceramics, and medical polymers) primarily relies on extensive animal experiments to obtain data 166. However, due to species differences, the data obtained by sacrificing animals often show inconsistencies in clinical trials 167. The construction of bone organoids allows for *in vitro* testing of bone implant materials, thereby replacing *in vivo* evaluation and reducing the use of animals. Human-derived bone organoids can better simulate the internal human environment, avoiding data inconsistencies caused by species differences, and enabling accurate preclinical evaluation of bone implant materials 15.

**Expert consensus 8:** Bone organoids should be prioritized as the primary platform for evaluating the biological effects of bone implants. Optimizing and standardizing the culture techniques of bone organoids, while strengthening the correlation between organoid models and clinical outcomes, will enhance the reliability and translational relevance of these models in clinical applications.

### 4.5 Precision medicine

As the future direction of medicine, precision medicine benefits from bone organoids that can simulate the bone tissue microenvironment while retaining their morphology, genome, and gene expression characteristics. This provides realistic drug response data, facilitating personalized drug screening and toxicity prediction, thereby improving the accuracy and efficiency of treatment. Studies have shown that patient-derived cancer organoids can simulate tumor genotypes and phenotypes, preserving tumor heterogeneity and intercellular interactions, which enhances the precision and efficiency of personalized cancer therapy and improves clinical treatment outcomes 168.

**Expert consensus 9:** Personalized bone organoid models should be constructed based on individual patient genomic and phenotypic characteristics to more accurately replicate disease conditions. Comprehensive studies on drug mechanisms within these models can help identify critical molecular targets and signaling pathways. Additionally, developing and validating bone disease biomarkers will allow for more precise prediction of patient treatment responses through the use of bone organoid testing.

## 5. How to identify bone organoids?

**Figure 5:**
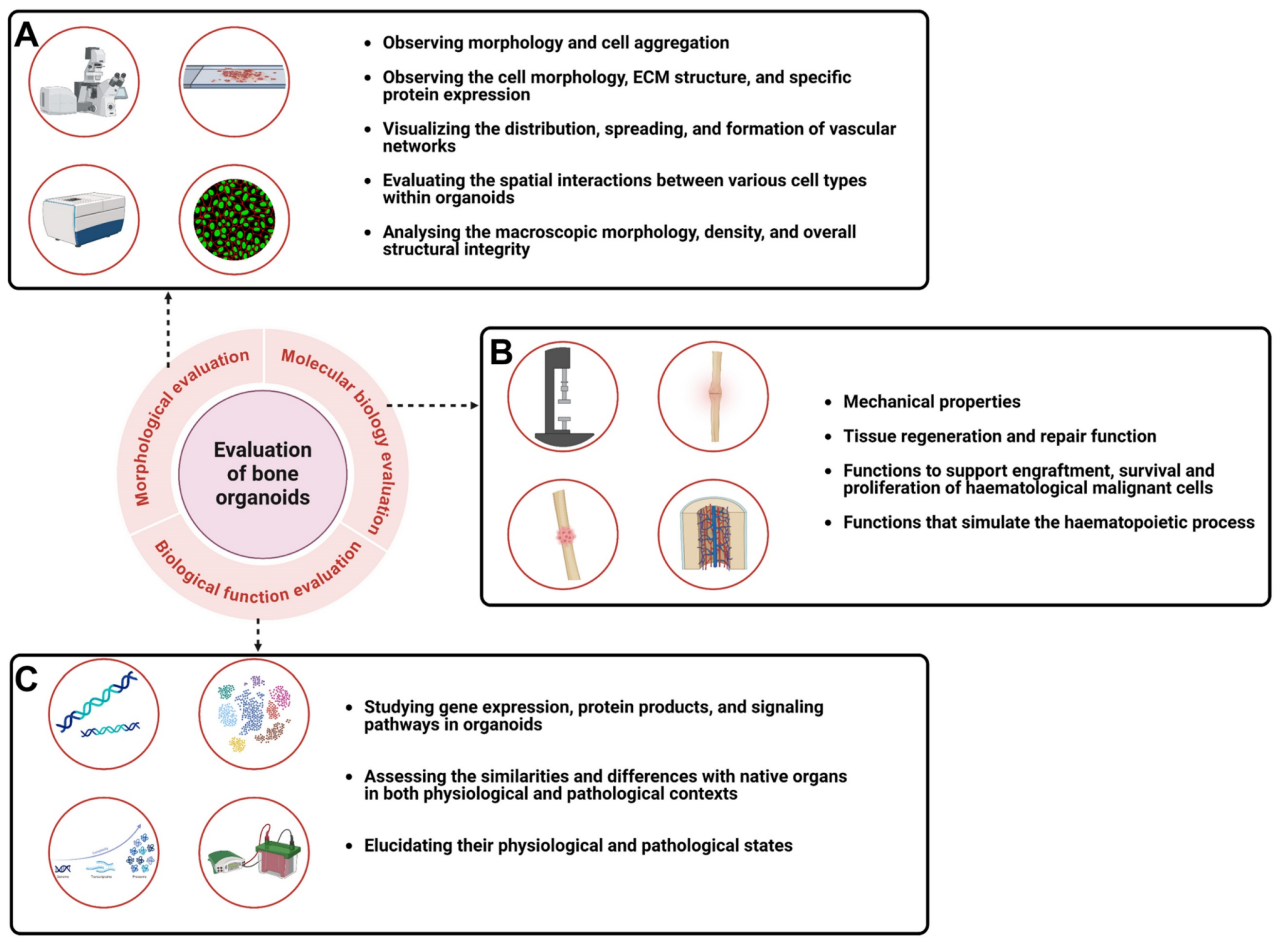
** Multidimensional and comprehensive identification of bone organoids. (A)** Morphological identification of bone organoids.** (B)** Biological function identification of bone organoids. **(C)** Molecular biology identification of bone organoids.

The identification and evaluation of bone organoids require a comprehensive analysis at multiple levels to meet research and clinical standards 13. Morphological assessment focuses on structural integrity and resemblance to natural bone tissue. Functional identification determines whether the organoids support bone regeneration, mineralization, and mechanical load-bearing. Molecular biology assessment analyzes gene expression, protein products, and signaling pathways to evaluate the physiological and pathological similarities between the organoids and natural tissues **(Figure 5)**. These combined assessments confirm that the bone organoids are structurally stable and functionally effective, making them suitable for therapeutic or experimental use.

### 5.1 Morphological identification

Morphological identification involves using various imaging techniques to assess the structural characteristics of bone organoids. Microscopy methods, such as light microscopy and scanning electron microscopy, are employed to observe the microstructure, including tissue morphology, cell distribution, and matrix composition 169. Continuous observation of bone organoid morphology and cell aggregation during culture using optical microscopy can assess the degree of self-assembly and differentiation maturity 35. Tissue section staining techniques, which involve the binding of dyes to tissue or cellular components, reveal various microstructures within the tissue cells. Embedding, sectioning, and applying specific staining techniques (such as H&E staining, Masson's trichrome staining, and immunohistochemistry staining) to bone organoids allow for the observation of cell morphology, ECM structure, and specific protein expression. Tissue clearing and 3D immunofluorescence imaging techniques can visualize the distribution, spreading, and formation of vascular networks within bone organoids 26, 37. Additionally, fluorescence scanning and multiplex quantitative mapping techniques can be used to evaluate the spatial interactions between various cell types within organoids 68. 3D imaging techniques like micro-CT scanning or micro-MRI are utilized for 3D reconstruction, allowing for a detailed analysis of the organoids' macroscopic morphology, density, and overall structural integrity 170, 171.

### 5.2 Biological function identification

Functional identification of bone organoids involves a series of tests to determine their suitability and effectiveness for potential *in vivo* applications 172. The human skeletal system performs various functions, including mechanical support, hematopoiesis, endocrine regulation, and calcium-phosphorus metabolism. Mechanical properties refer to the physical characteristics exhibited when subjected to force, including strength, hardness, elasticity, and toughness 173, 174. Unlike other organs, bones are rich in inorganic minerals, which endow them with excellent mechanical support and the ability to withstand mechanical stress. The mechanical properties not only affect the morphology and structural stability of bone-related organoids but are also associated with their physiological and pathological states, making them an important indicator for evaluating bone organoids. Bone organoids should possess excellent mechanical properties, with a Young's modulus approaching that of natural cancellous bone 26. Under mechanical stimulation, the hardness and mineral density of bone organoids increase 132. When trabecular bone organoids are cultured in a microgravity environment, they can replicate pathological bone loss and the imbalance of bone remodeling* in vitro* 33. Tissue regeneration and repair are the main biological functions of organoids. Bone callus organoids transplanted into femoral defects have demonstrated potential for promoting bone regeneration 60. Bone marrow organoids support the engraftment, survival, and proliferation of hematologic malignancy cells. Tumor cells derived from patients with multiple myeloma and chronic myeloid leukemia, when cultured in bone marrow organoids, have shown that these organoids can provide a suitable environment to support tumor cell growth 37. Furthermore, composite bone marrow organoids can generate arterial endothelial cells and hematopoietic endothelial cells that simulate the hematopoietic process 36.

### 5.3 Molecular biology identification

Molecular biology techniques are employed to investigate gene expression, protein production, and signaling pathways within organoids 175. By analyzing the molecular characteristics of organoids, researchers can assess their similarities and differences with native organs in both physiological and pathological contexts 11. This evaluation includes omics analysis and various molecular biology techniques. Omics analysis encompasses genomics, transcriptomics, proteomics, and metabolomics, which reveal the complex biological processes of organoids by examining their molecular features. Molecular biology techniques such as qPCR, Western blotting, immunofluorescence, and RNA sequencing are employed to specifically detect gene and protein expression levels in organoids, thereby elucidating their physiological and pathological states 176, 177. Klein et al. constructed gene-deficient bone marrow organoids and demonstrated their ability to model monogenic bone marrow diseases 36. Transcriptome sequencing, through analysis at multiple time points, elucidates the complex molecular regulatory mechanisms of bone organoids during the bone healing process 26. Vail et al. employed small RNA sequencing to map the transcriptome of cartilage organoids 178. Papantoniou et al. confirmed that the temporal gene expression patterns during callus organoid formation follow the process of endochondral ossification through transcriptome sequencing 35. Similarly, In the study of callus organoid formation, transcriptome sequencing revealed the temporal sequence of gene expression related to endochondral ossification and confirmed that callus organoids possess bone-cartilage lineage differentiation potential 35, 60. Advanced techniques such as single-cell RNA sequencing (scRNA-seq) and spatial transcriptomics enable a more precise evaluation of the transcriptional differences among different cell types within organoids over time and space. Using scRNA-seq technology, researchers confirmed that the hematopoietic and stromal cell lineages in bone marrow organoids exhibit a high degree of transcriptional homology with those in humans 36, 37. Currently, bone organoids exhibit relatively simple structures and functions, with limited protein diversity and metabolic activity 179. Genomics, transcriptomics, and conventional molecular biology techniques suffice for evaluating bone organoids. However, as research progresses, proteomics and metabolomics will provide detailed insights into the biological functions, signaling pathways, metabolic states, and energy conversions within bone organoids. These approaches will help decode the protein interactions and metabolic processes that drive bone organoid development and function.

**Expert consensus 10:** It is recommended to assess the morphology of bone organoids using techniques such as optical microscopy, histopathology, 3D immunofluorescence imaging, and multiplex quantitative mapping. Functional identify should focus on key biological aspects, including mechanical properties, hematopoietic function, endocrine activity, and calcium-phosphorus metabolism. Molecular assessments can be conducted through genomics, transcriptomics, proteomics, and metabolomics to analyze genetic information and metabolic products. For biosafety evaluation, cell and/or animal models should be employed, depending on factors such as cell sources, matrix materials, and inductive factors. Additionally, AI-driven image analysis tools, combined with common morphological parameters (e.g., area, volume, cell arrangement), are recommended to introduce specific quantitative methods, enabling consistent data comparisons across laboratories. AI can automate the processing and analysis of large image datasets, significantly enhancing the efficiency and accuracy of quantitative analyses, thereby standardizing evaluation procedures, ensuring comparability and reproducibility under different experimental conditions, and ultimately improving the scientific rigor and precision of bone organoid evaluations.

## 6. Challenges and prospects

The construction of bone organoids faces numerous challenges, primarily due to the complex structure of bone tissue and its unique physiological demands. First, the intricate multicellular microenvironment and the mechanical stress-bearing bone matrix make it extremely challenging to construct complete bone structures *in vitro*. Current constructions of bone organoids typically focus on specific functional structures of bone tissue, such as mineralization, bone marrow, trabeculae, and woven bone 26, 27, 34, 68. Constructing integrated bone organoids that include both cortical and cancellous bone along with bone marrow remains a critical challenge to overcome. Second, vascularization is a key difficulty in the construction of bone organoids. The ability to vascularize is fundamental to ensuring that bone organoids can be cultured long term and reach clinical applications. The complex vascular network in bones is tightly coupled with osteogenesis 180. Issues with vascularization leading to insufficient nutrient supply restrict the size and clinical application of bone organoids 181. Additionally, existing bone organoid models lack multi-system coordination capabilities. Bone development involves the coordinated actions of multiple systems, including immune, endocrine, and nervous systems, yet current organoid models do not effectively reproduce these interactions. It is believed that with the rapid development of organ-on-a-chip technology, effective strategies might be provided to address this issue 182.

As an emerging biomedical research model, bone organoids hold broad application prospects in fields such as disease research, drug screening, and regenerative medicine. This consensus aims to provide a set of scientific and standardized guidelines and frameworks to promote the healthy and orderly development of bone organoids. With continuous technological advancements and deeper research, bone organoids will demonstrate their unique advantages and value in more fields. Future research on bone organoids should not only focus on basic research but also on clinical applications. By leveraging the bioeffects of materials and integrating AI technology, the bottlenecks in constructing bone organoids can be addressed. Given the complexity of the skeletal system, achieving fully functional composite bone organoids in the short term is highly challenging, thus requiring a phased strategy to gradually achieve this goal. Initially, the physiological functions of normal bone should be simulated, followed by the replication of pathological features of bone diseases, further replicating micro and macro structures, and ultimately achieving the mass and consistent production of fully functional bone organoids. Through continuous technological innovation and multidisciplinary collaboration, bone organoids are expected to become an important tool in future medical research, further enhancing our understanding and treatment capabilities for bone diseases.

## Supplementary Material

Supplementary methods.

## Figures and Tables

**Table 1:** Comparison of seed cells for organoid development

Cell type	Source	Characteristics	Advantages	Commonly used for
MSCs	Bone marrow, adipose tissue, umbilical cord	Multipotent, can differentiate into bone, cartilage, and fat cells	High proliferative capacity, ease of isolation, ability to form various tissues	Bone, cartilage, and adipose tissue organoids
iPSCs	Reprogrammed somatic cells	Pluripotent, can differentiate into any cell type	High differentiation potential, ability to create patient-specific models	Disease modeling, personalized medicine, complex organoid construction
Osteoblast precursor cells	Bone marrow, periosteum	Osteogenic potential, capable of differentiating into osteoblasts	Direct contribution to bone formation, more specific to bone tissue	Bone organoids, bone regeneration models
Endothelial cells	Blood vessels, umbilical vein	Can form blood vessel-like structures, important for vascularization	Key for creating vascularized organoids, essential for long-term culture	Vascularized bone organoids, tissue engineering
Chondrocytes	Cartilage tissue	Can form cartilage and ECM	Directly relevant for cartilage formation, good for joint disease models	Cartilage organoids, joint disease research
ASCs	Adipose tissue	Multipotent, similar to MSCs but with easier access and higher yield	Non-invasive collection, abundant source	Bone, cartilage, and adipose tissue organoids
Fibroblasts	Skin, connective tissues	Can be reprogrammed into induced iPSCs	Widely available, easy to culture	Modeling connective tissue diseases, reprogramming to iPSCs

**Table 2:** Comparison of matrix materials for organoid construction

Matrix material	Source	Characteristics	Advantages	Commonly used for
Collagen	Animal tissues (e.g., rat, bovine)	Biodegradable, supports cell adhesion, mimics extracellular matrix	Biocompatible, widely used for various tissue types	Bone, cartilage, and soft tissue organoids
Matrigel	Derived from Engelbreth-Holm-Swarm (EHS) mouse sarcoma	A gel-like mixture of ECM proteins	High bioactivity, supports various cell types	Breast cancer organoids, stem cell culture
Hydrogel (e.g., PEG-based)	Synthetic polymers	Hydrophilic, customizable stiffness and degradation rates	Tunable mechanical properties, mimics tissue elasticity	Bone, cartilage, and soft tissue organoids, wound healing models
Alginate	Brown seaweed	Ionic gel, biocompatible, forms hydrogels in the presence of calcium ions	Biodegradable, easy to handle and process	Cartilage organoids, wound healing applications
Fibrin	Derived from fibrinogen (human plasma)	Forms gel in presence of thrombin and calcium	Supports cell migration and tissue growth, biodegradable	Vascularized organoids, tissue engineering
Decellularized ECM	Animal tissues (e.g., heart, liver)	Retains the native structure and bioactive components of the ECM	Preserves native cell-matrix interactions, highly bioactive	Heart, liver, and kidney organoids, tissue scaffolds
Poly(lactic-co-glycolic acid) (PLGA)	Synthetic copolymer	Biodegradable, provides controlled drug release properties	Versatile, tunable degradation rates, supports bone tissue formation	Bone regeneration, drug delivery systems
Chitosan	Chitin (derived from shellfish)	Biocompatible, antimicrobial, can form gels with variable stiffness	Natural material, supports cell adhesion, biodegradable	Bone and cartilage organoids, tissue scaffolds
Silk fibroin	Silk from silkworms	Biocompatible, strong mechanical properties, tunable degradation rates	Strong, flexible, supports cell growth	Bone tissue engineering, vascular scaffolds

## Abbreviations

3D: three-dimensional
2D: two-dimensional
ESCs: embryonic stem cells
iPSCs: induced pluripotent stem cells
HSCs: hematopoietic stem cells
PSCs: pluripotent stem cells
MSCs: mesenchymal stem cells
HSPCs: hematopoietic stem and progenitor cells
BMSCs: Bone marrow mesenchymal stem cells
GelMA: gelatin methacrylate
DPSCs: Dental pulp stem cells
SSCs: skeletal stem/progenitor cells
ASCs: adipose-derived stem cells
BMMs: bone marrow mononuclear cells
DBP: demineralized bone paper
ECM: extracellular matrix
EHS: Engelbreth-Holm-Swarm
AlgMA: alginate methacrylate
PEG: polyethylene glycol
EVs: extracellular vesicles
dECM: decellularized extracellular matrix
PLGA: poly (lactic-co-glycolic acid)
Ceffe: cell-free fat extract
PLLA: polylevolactic acid
DLP: digital light processing
AI: artificial intelligence
HAP: hydroxyapatite
BMP4: morphogenetic protein 4
FGF2: fibroblast growth factor, VEGFA: vascular endothelial growth factor A
SCF: stem cell factor
FLT3: FMS-like tyrosine kinase 3
IL3: interleukin-3
IL6: interleukin-6
G-CSF: granulocyte colony-stimulating factor
EPO: erythropoietin
TPO: thrombopoietin
IL-1β: interleukin-1 beta
TNF-α: tumor necrosis factor-alpha
OPG-XL: osteoprotegerin (OPG) with additional 19 amino acids at its C-terminus
OA: osteoarthritis
scRNA-seq: single-cell RNA sequencing
