# Supplementary material for: Standardization and consensus in the development and application of bone organoids
Source: Theranostics. 2025 Jan 1;15(2):682–706. doi: 10.7150/thno.105840 (PMC11671374; doi:10.7150/thno.105840)
Supplement: Supplementary file 1 — Supplementary methods. [file thnov15p0682s1.pdf]

Standardization and consensus in the development and application of bone organoids

Jian Wang[1,2,3,4,5*], Xiao Chen[1,2,3,4,5*], Ruiyang Li[1,2,3*], Sicheng Wang[6*], Zhen Geng[4,5], Zhongmin Shi[7], Yingying Jing[4,5], Ke Xu[4,5], Yan Wei[4,5], Guangchao Wang[1,2,3], Chongru He[1,2,3], Shiwu Dong[8], Guohui Liu[9], Zhiyong Hou[10], Zhidao Xia[11], Xinglong Wang[12], Zhou Ye[13], Fengjin Zhou[14#], Long Bai[4,5#], Hongbo Tan[15#], Jiacan Su[1,2,3,4,5#]

1 Department of Orthopedics, Xinhua Hospital Affiliated to Shanghai Jiao Tong University School of Medicine, Shanghai, 200092, China

2 Trauma Orthopedics Center, Xinhua Hospital Affiliated to Shanghai Jiao Tong University School of Medicine, Shanghai 200092, China

3 Institute of Musculoskeletal Injury and Translational Medicine of Organoids, Xinhua Hospital Affiliated to Shanghai Jiao Tong University School of Medicine, Shanghai, 200092, China

4 Institute of Translational Medicine, Shanghai University, Shanghai, 200444, China

5 National Center for Translational Medicine SHU Branch, Shanghai University, Shanghai, 200444, China

6 Department of Orthopedics, Shanghai Zhongye Hospital, Shanghai, 200941, China

7 Sixth People's Hospital Affiliated to Shanghai Jiao Tong University School of Medicine, Shanghai 200233, China

8 Department of Biomedical Materials Science, College of Biomedical Engineering, Third Military Medical University, Chongqing, 400038, China.

9 Union Hospital Affiliated to Tongji Medical College, Huazhong University of Science and Technology, Wuhan 430022, China

10 Department of Orthopaedic Surgery, Third Hospital of Hebei Medical University, Shijiazhuang, 050051, China

11 Institute of Life Science, College of Medicine, Swansea University, Swansea, SA2 8PP, UK

12 Department of Pharmacology and Toxicology, University of Arizona, Tucson, AZ, 85721， USA

13 Applied Oral Sciences and Community Dental Care, Faculty of Dentistry, The University of Hong Kong, Hong Kong, 999077, China

14 Department of Orthopedics, Honghui Hospital, Xi'an Jiao Tong University, Xi'an, 710000, China

15 Department of Orthopedics, 920th Hospital of Joint Logistics Support Force of Chinese PLA, Kunming， 650032, China

[*]These authors contributed equally to this work.

[#]Corresponding authors.

E-mail addresses: dr.zhoufj@163.com (F. Zhou), bailong@shu.edu.cn (L. Bai), tanhongbo021@yeah.net (H. Tan), drsujiacan@163.com (J. Su).

Supplementary Methods

1. Expert invitation

To ensure a comprehensive and interdisciplinary approach to the consensus on bone organoid development and application, we invited experts from various fields including stem cell biology, tissue engineering, bone biology, biomaterials, and clinical orthopedics. Experts were selected based on their significant contributions to the fields of organoid technology and bone tissue engineering, with a focus on their publication

history in high-impact journals and experience in bone-related research and clinical applications. The selection criteria emphasized expertise in bone physiology, osteogenesis, and regenerative medicine, ensuring that the consensus would have clinical relevance. Additionally, diversity in research backgrounds and geographical representation was prioritized to foster a global and inclusive perspective. Invitations were extended to senior researchers, early-career scientists, and clinicians, all of whom contributed their unique insights into the challenges and opportunities in bone organoid technology. Experts could nominate additional researchers, with final selections made by the core team (Fengjin Zhou, Long Bai, Hongbo Tan, Jiacan Su). This expert group participated in a series of virtual meetings and discussions, providing valuable input that shaped the final consensus, which aims to guide future research and clinical translation in the field of bone organoids.

2. Consensus building

The consensus-building process was designed to gather expert opinions on the development and application of bone organoids, ensuring both scientific rigor and clinical relevance. It began with the core team drafting a detailed questionnaire covering critical topics such as cell types, matrix materials, and construction methods for bone organoids. Experts were invited to provide quantitative and qualitative feedback, which was discussed in a series of virtual roundtable meetings. These discussions allowed experts to present their views, debate approaches, and propose new ideas, while the core team moderated and refined the questionnaire based on feedback. Consensus was defined as ≥90% agreement on key questions, and where disagreements arose, follow-up discussions were held to address concerns. After the meetings, a summary was circulated, and a revised version of the questionnaire was distributed for final review. The process culminated in a formal consensus report, outlining the agreed standards for bone organoid research and providing a framework for future studies.
